# A High-Throughput Forward Genetic Screen Identifies Genes Required for Virulence of *Pseudomonas syringae* pv. *maculicola* ES4326 on Arabidopsis

**DOI:** 10.1371/journal.pone.0041461

**Published:** 2012-08-01

**Authors:** Karl J. Schreiber, David Ye, Eric Fich, Allen Jian, Timothy Lo, Darrell Desveaux

**Affiliations:** 1 Department of Cell and Systems Biology, University of Toronto, Toronto, Ontario, Canada; 2 Centre for the Analysis of Genome Evolution and Function, University of Toronto, Toronto, Ontario, Canada; Niels Bohr Institute, Denmark

## Abstract

Successful pathogenesis requires a number of coordinated processes whose genetic bases remain to be fully characterized. We utilized a high-throughput, liquid media-based assay to screen transposon disruptants of the phytopathogen *Pseudomonas syringae* pv. *maculicola* ES4326 to identify genes required for virulence on Arabidopsis. Many genes identified through this screen were involved in processes such as type III secretion, periplasmic glucan biosynthesis, flagellar motility, and amino acid biosynthesis. A small set of genes did not fall into any of these functional groups, and their disruption resulted in context-specific effects on *in planta* bacterial growth.

## Introduction

Historically, forward genetic screens have provided valuable insights into the various regulatory elements, chemicals, and other cellular components required for the virulence of bacterial phytopathogens [1]–[5]. With few exceptions, however (e.g. [6]), these screens have generally been relatively low-throughput and labor-intensive. We previously developed an assay in which *Arabidopsis thaliana* (hereafter Arabidopsis) seedlings were grown in liquid media in 96-well plates [7]. Inoculation of wells with *Pseudomonas syringae* resulted in the eventual bleaching of seedling cotyledons, and this phenotype was directly related to the virulence of these strains. In this study, we generated a collection of transposon disruptants in *P*. *syringae* pv. *maculicola* ES4326 (*Pma* ES4326), which is virulent on both Arabidopsis and radish. We subsequently used our high-throughput assay to screen this collection in order to identify genes required for bacterial virulence. Importantly, this type of large-scale survey has not previously been performed with *Pma* ES4326. There is significant diversity amongst different *P. syringae* isolates with regards to type III-secreted effector gene repertoires [8], suggesting wide variation in virulence strategies and highlighting the value of searching for new virulence-associated genes in different members of the *P. syringae* phylogeny. Indeed, our work provided novel insights into the nutritional requirements of *Pma* ES4326 in Arabidopsis and revealed genes not previously known to be associated with *P. syringae* virulence.

## Materials and Methods

### High-throughput Screening for Transposon Disruptants with Reduced Virulence

Transposon disruptants were generated using a mini-Tn*5* (mTn*5*) transposon bearing a kanamycin resistance gene [9] which was introduced into *Pma* ES4326 by triparental mating [10]. Disruptants were recovered on selective media (King’s B [11] plus 300 µg/mL streptomycin and 50 µg/mL kanamycin) incubated at 28°C. Arabidopsis (ecotype Col-0) seedlings were grown in liquid media (0.5X Murashige and Skoog (MS) basal salts, 2.5 mM 2-(N-Morpholino)ethanesulfonic acid (MES), pH 5.8) as described previously [7]. After five days of growth, wells were inoculated with the disruptants at 1×10^5^ cfu/mL (one disruptant per well) and seedling phenotypes assessed after seven days of incubation on a vibrating shaker at 22°C under continuous light. Experiments were conducted in triplicate, and those disruptants that failed to cause the bleaching of seedling cotyledons in all three replicates were considered hits. Bacterial populations in the liquid media of hit wells were quantified by dilution plating as a preliminary assessment of potential auxotrophy. Bacterial growth was also measured in King’s B media in order to exclude hits arising from general metabolic defects. Disruptants whose *in vitro* growth in both media resembled that of wildtype *Pma* ES4326 were retested in the liquid assay to confirm the reduced virulence phenotype. Following validation, the genomic location of each transposon was determined using vectorette PCR [12].

### *In vitro* Characterization of Transposon Disruptants

Bacterial growth was monitored in rich media (King’s B), *Pseudomonas* minimal media supplemented with 10 mM fructose (PMMF) [13], and MS media in which seedlings had been grown (MSS). Bacteria grown on agar plates were first resuspended in PMMF, adjusted to OD_600_ = 0.01 in the appropriate media, then distributed to 96-well plates. Covered plates were incubated with shaking at 28°C and samples withdrawn at regular intervals for quantitation of bacterial populations by dilution plating.

For gene expression analyses, culture conditions, RNA extraction methods, and real-time quantitative PCR parameters are described in Methods S1. Primers used are listed in Table S1.

### *In planta* Characterization of Transposon Disruptants

Bacterial growth was evaluated in five- to six-week-old Arabidopsis (Col-0) plants that had been cultivated in soil (ProMix BX, Premier Horticulture Ltd., Dorval, PQ, Canada) amended with 20-20-20 fertilizer, grown in a controlled environment room with a nine-hour photoperiod and a day/night temperature regime of 22°C/18°C. To monitor apoplastic bacterial growth, plants were inoculated by pressure infiltration using a needleless syringe and an inoculum concentration of OD_600_ = 0.0002 (1×10^5^ cfu/mL) prepared in 10 mM MgCl_2_. Spray inoculations were also performed in order to evaluate tissue entry (dependent upon both epiphytic survival and the suppression of stomatal innate immune responses) as well as subsequent apoplastic growth by *Pma* ES4326. For these inoculations, bacterial suspensions were adjusted to OD_600_ = 0.8 (4×10^8^ cfu/mL) in 10 mM MgCl_2_ containing 0.02% Silwet L-77 surfactant (GE Silicones, South Charleston, WV, USA). Inocula were sprayed on adaxial leaf surfaces to the point of runoff using a Preval aerosol sprayer (Babco Sales Ltd, Surrey, BC, Canada). Plants were covered with a clear plastic dome for 24 hours after spraying in order to maintain conditions of high humidity. In all experiments, bacterial growth within inoculated tissues was quantified at three days post-inoculation by dilution plating of tissue homogenates as described previously [14], although tissue samples from spray inoculations were surface-sterilized with 70% ethanol for 30 sec, then washed with sterile distilled water for 30 sec prior to this analysis.

## Results and Discussion

### Type III Secretion System Genes

**Figure 1:**
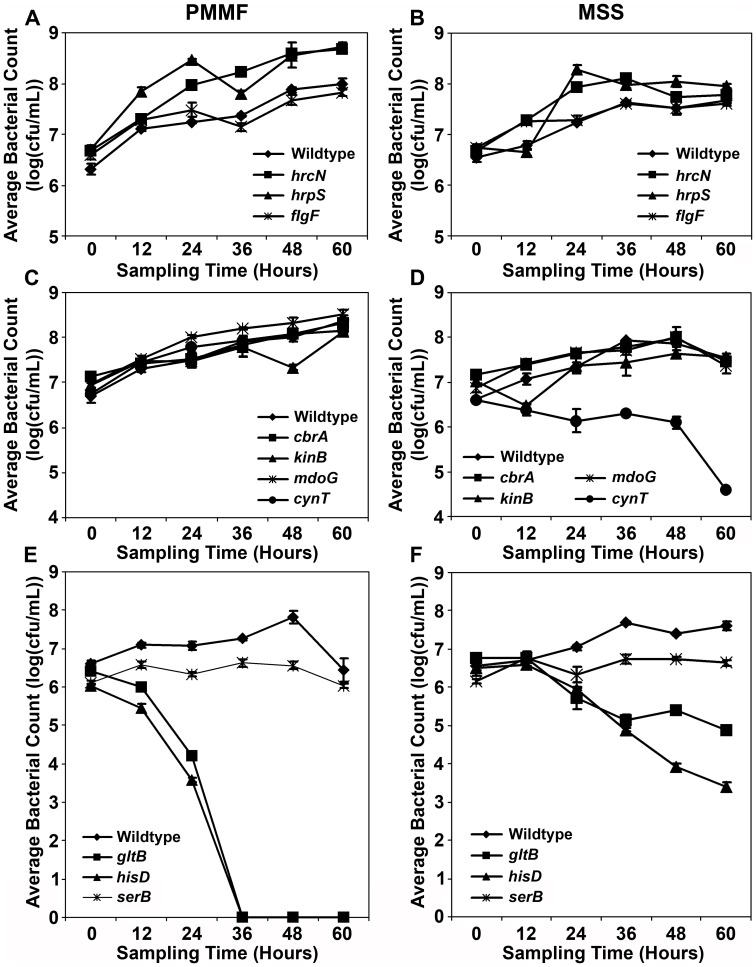
*In vitro* growth of *Pma* ES4326 transposon disruptants in *Pseudomonas* minimal media containing 10 mM fructose (PMMF) (A, C, E) or MS media in which Arabidopsis seedlings had been grown (MSS) (B, D, F). Cultures were adjusted to OD_600_ = 0.01 and incubated with shaking at 28°C. Bacterial populations were quantified at 12-hour intervals by dilution plating. With the exception of wildtype *Pma* ES4326, genotype names indicate the gene that is disrupted by a mini-Tn*5* transposon. Error bars reflect standard deviation of the mean of four replicate samples. Three independent experiments were performed with similar results.

Approximately 12,600 disruptants were screened in the liquid assay, yielding 40 hits (Table 1). Nearly half of these hits involved genes associated with the type III secretion system (TTSS), including structural components (*hrcC*, *hrcJ*, *hrcQb*, *hrpQ*, *hrcS*, *hrcU*, *hrcV*), enzymes (*hrpB*, *hrcN*, *hrpO*), and upstream regulatory elements that control *hrp*/*hrc* gene transcription (*hrpG*, *hrpR*, *hrpS*). The importance of the TTSS for bacterial virulence is well established [15], and we previously observed significant impairments of *in planta* growth (1–2 logs) by TTSS-inactive disruptants of *Pma* ES4326 ([16]; K.J. Schreiber and D. Desveaux, unpublished data). Given the large number of genes encoding TTSS components and the dramatic effects of their disruption, the recovery of numerous TTSS gene disruptants could be expected. Of note was the recovery of five independent disruptions of the TTSS ATPase gene *hrcN*, contrasting with only one disruptant for the vast majority of genes identified through the screen. While the Tn*5* transposon does exhibit some selectivity in its insertion into plasmids [17], [18], factors affecting the insertion of the mini-Tn*5* derivative into larger genomes remain to be evaluated. Also notable was the slightly increased growth of the TTSS gene disruptants relative to wildtype *Pma* ES4326 in both PMMF and MSS media (Fig. 1A, B). A nonfunctional TTSS may confer a metabolic benefit to the bacteria in this context if fewer resources are directed to type III secretion machinery overall, similar to recent observations regarding *Salmonella* growth *in vitro* [19].

**Table 1:** *Pseudomonas syringae* pv. *maculicola* ES4326 transposon disruptants with reduced virulence in a high-throughput liquid media-based assay.

Group^a^	Gene Affected	Number Identified	(Putative) Protein Function	Location Within Operon
**I**	*hrcN*	5	ATPase	*hrpJ-hrcV-hrpQ-* ***hrcN*** *-hrpO-hrpP-hrcQa-hrcQb-hrcR-hrcS-hrcT-hrcU*
	*hrcQb*	2	type III secretion-associated protein	*hrpJ-hrcV-hrpQ-hrcN-hrpO-hrpP-hrcQa-* ***hrcQb*** *-hrcR-hrcS-hrcT-hrcU*
	*hrpS*	1	TTSS transcriptional regulator	*hrpR-* ***hrpS***
	*hrpG*	1	TTSS regulatory protein	*hrpF-* ***hrpG*** *-hrcC-hrpT-hrpV*
	*hrpQ*	1	inner membrane structural TTSS component	*hrpJ-hrcV-* ***hrpQ*** *-hrcN-hrpO-hrpP-hrcQa-hrcQb-hrcR-hrcS-hrcT-hrcU*
	*hrpB*	1	ATP-dependent helicase	*hrpZ1-* ***hrpB*** *-hrcJ-hrpD*
	*hrcJ*	2	lipoprotein; inter-membrane bridge	*hrpZ1-hrpB-* ***hrcJ*** *-hrpD*
	*hrcU*	1	TTSS inner membrane component	*hrpJ-hrcV-hrpQ-hrcN-hrpO-hrpP-hrcQa-hrcQb-hrcR-hrcS-hrcT-* ***hrcU***
	*hrpO*	1	regulator of TTSS protein export	*hrpJ-hrcV-hrpQ-hrcN-* ***hrpO*** *-hrpP-hrcQa-hrcQb-hrcR-hrcS-hrcT-hrcU*
	*hrcS*	1	TTSS structural component	*hrpJ-hrcV-hrpQ-hrcN-hrpO-hrpP-hrcQa-hrcQb-hrcR-* ***hrcS*** *-hrcT-hrcU*
	*hrcV*	1	TTSS structural component	*hrpJ-* ***hrcV*** *-hrpQ-hrcN-hrpO-hrpP-hrcQa-hrcQb-hrcR-hrcS-hrcT-hrcU*
	*hrpR*	1	TTSS transcriptional regulator	***hrpR*** *-hrpS*
	*hrcC*	1	outer membrane TTSS protein	*hrpF-hrpG-* ***hrcC*** *-hrpT-hrpV*
**II**	*fleN*	1	negative regulator of flagellar number	***fleN*** *-fliA*
	*fliH*	2	putative flagellar assembly protein	*fliE-fliF-fliG-* ***fliH*** *-fliI-fliJ*
	*flgF*	1	flagellar basal-body rod protein	***flgF*** *-flgG-flgH-flgI-flgJ-flgK-flgL*
	*fliG*	1	flagellar motor switch protein	*fliE-fliF-* ***fliG*** *-fliH-fliI-fliJ*
	*flgH*	1	flagellar L-ring protein	*flgF-flgG-* ***flgH*** *-flgI-flgJ-flgK-flgL*
**III**	*galU*	1	UTP-glucose-1-phosphate uridylyltransferase	single gene
	*mdoG*	1	periplasmic glucan biosynthesis	***mdoG*** *-mdoH*
	*mdoH*	1	periplasmic glucan biosynthesis	*mdoG-* ***mdoH***
**IV**	*gltB*	3	glutamate synthase (large subunit)	single gene
	*hisD*	2	histidinol dehydrogenase - histidine biosynthesis	***hisD*** *-hisC*
	*serB*	1	phosphoserine phosphatase - serine biosynthesis	single gene
**V**	*cynT*	3	carbonic anhydrase	single gene
	*cbrA*	1	sensory box histidine kinase	***cbrA*** *-cbrB*
	*kinB*	1	sensory box histidine kinase	*algB-* ***kinB***

a Disruptants are grouped according to the known or predicted functions of the disrupted genes as follows: type III secretion (Group I), flagellar motility (Group II), periplasmic glucan biosynthesis (Group III), amino acid biosynthesis (Group IV), or other metabolic processes (Group IV).

### Flagellar Motility Genes

Bacterial flagella share many structural similarities with the TTSS [20], and are essential appendages for swimming motility [21]. We identified six disruptants representing five different genes, four of which (*flgF*, *flgH*, *fliG*, *fliH*) encode structural flagellar components (Table 1). The disruption of *fleN*, which encodes a negative regulator of flagellar abundance, also dramatically reduced expression of the downstream gene *fliA* (Fig. S1). FliA is an alternative sigma factor (σ^28^) that regulates genes associated with the late stages of flagellar assembly [22], such that the loss of *fliA* expression in a *fleN* disruptant results in a largely nonmotile bacterium. In fact, all of the flagellar gene disruptants recovered in this screen were nonmotile (Fig. S2), suggesting that flagellar motility is important for bacterial entry into host tissues. Indeed, although the *in planta* growth of nonmotile disruptants was comparable to that of wildtype *Pma* ES4326 when inoculated into soil-grown plants by pressure infiltration, significant reductions were observed following spray inoculation [16]. *In vitro* growth was unaffected (Fig. 1A, B). In the liquid assay, the reduction in bleaching symptoms induced by the flagellar gene disruptants was more subtle than that observed for the TTSS gene disruptants, likely owing to the continuous contact between bacteria and seedlings for the entire experiment, which could allow some bacteria to passively enter plant tissues. Nonetheless, the impaired virulence of nonmotile transposon disruptants could be detected in the liquid assay using the seedling bleaching phenotype. It should be noted that additional flagellar gene disruptants were likely present in the disruptant collection, but hits shown to be nonmotile in a parallel motility screen [16] were not actively characterized in the later part of the virulence screen. Interestingly, ten such nonmotile disruptants were identified amongst the primary screening hits, so the total number of hits in this category is similar to the number of TTSS gene disruptants recovered from the screen.

### Periplasmic Glucan Biosynthesis Genes

**Figure 2:**
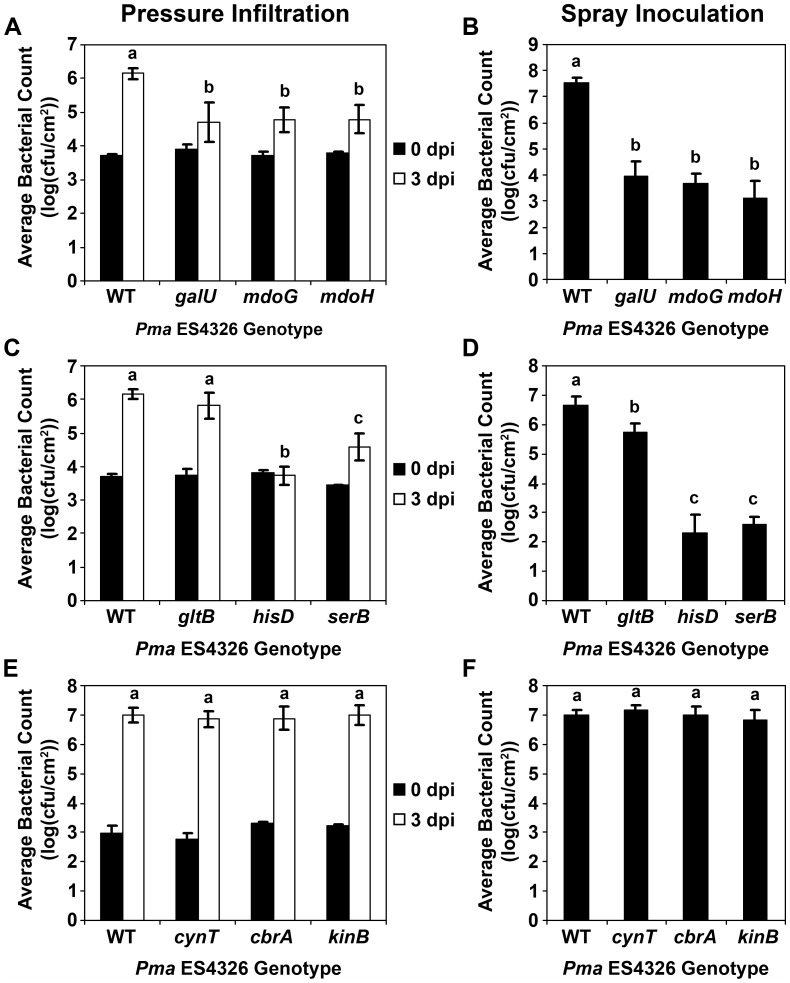
*In planta* growth of *Pma* ES4326 transposon disruptants in six-week-old soil-grown Arabidopsis plants, assessed by either pressure infiltration (A, C, E) or spray inoculation (B, D, F). Bacterial populations were quantified by dilution plating at zero and three days post-inoculation (dpi) for pressure infiltration and at three dpi for spray inoculation. With the exception of wildtype *Pma* ES4326 (WT), genotype names indicate the gene that is disrupted by a mini-Tn*5* transposon. Error bars reflect standard deviation of the mean of 10–12 replicate samples. Letters above data points indicate statistical significance groups as determined by pairwise Student’s t-tests (α = 0.05). At least two independent experiments were performed with similar results.

The cell walls of Gram-negative bacteria often contain membrane-derived oligosaccharides such as lipopolysaccharide which provide structural reinforcement as well as protection from extracellular chemical and osmotic stresses [23], [24]. One of the primary building blocks for these molecules is UDP-glucose [25], and we identified three disruptants of genes involved in either UDP-glucose synthesis (*galU*) or polymerization (*mdoG*, *mdoH*) (Table 1). The disruption of these genes in *P. syringae* has pleiotropic effects on processes such as biofilm formation, type III secretion, and motility, with dramatic negative consequences for epiphytic bacterial survival and endophytic growth [26], [27]. Such growth reductions were also observed in the *Pma* ES4326 disruptants (Fig. 2A, B), although *in vitro* growth was slightly higher than wildtype *Pma* ES4326 (Fig. 1C, D). Type III secretion was also impaired, as evidenced by the inability of these disruptants to elicit a TTSS-dependent programmed cell death response in the Eilenburg-0 ecotype of Arabidopsis (Fig. S3).

### Amino Acid Biosynthesis Genes

**Figure 3:**
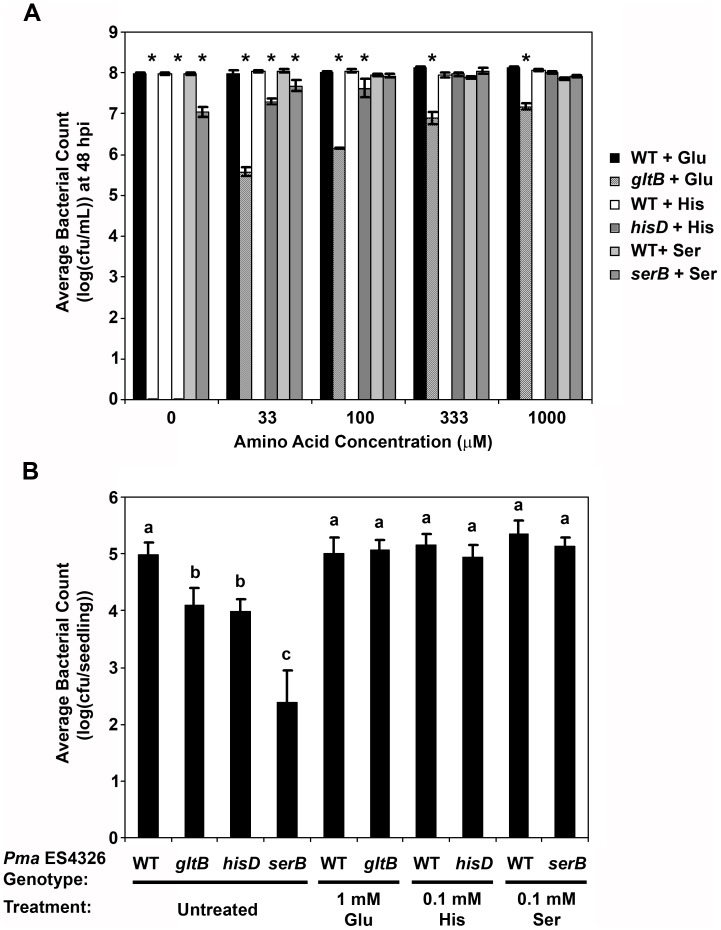
Rescue of *in vitro* and *in planta* growth defects of *Pma* ES4326 transposon disruptants by amino acid supplementation. For *in vitro* analyses, cultures were prepared with disruptants of genes associated with the biosynthesis of glutamate (*gltB*), histidine (*hisD*), and serine (*serB*), using *Pseudomonas* minimal media containing 10 mM fructose and supplemented with the appropriate amino acid (A). Cultures were adjusted to OD_600_ = 0.01, incubated with shaking at 28°C, and bacterial populations were quantified at 48 hours post-inoculation (hpi) by dilution plating. An asterisk indicates a statistically significant difference between wildtype (WT) *Pma* ES4326 and a given disruptant treated with the same amino acid (Student’s t-test, α = 0.05). For *in planta* virulence assays, five-day-old Arabidopsis seedlings grown in liquid media were also incubated with these disruptants in the presence or absence of exogenous amino acids (B). Bacterial growth within seedlings was quantified by dilution plating at three days post-inoculation. Error bars reflect standard deviation of the mean of four replicate samples. Letters above data points indicate statistical significance groups as determined by pairwise Student’s t-tests (α = 0.05). Three independent experiments were performed with similar results.

Three hits from the screen contained transposons in genes that contribute to the biosynthesis of glutamate (*gltB*), histidine (*hisD*), or serine (*serB*) (Table 1). *In vitro*, the *serB* disruptant exhibited only slight growth in PMMF, while populations of the *hisD* and *gltB* disruptants rapidly collapsed in this media (Fig. 1E). This collapse was mitigated somewhat in MSS media (Fig. 1F), although populations still decreased over time. In the original liquid assay screening plates, bacterial populations of these disruptants in the media were generally equivalent to wildtype *Pma* ES4326 at six to seven days post-inoculation, suggesting that amino acids were supplied by the seedlings in liquid media. Notably, PMMF supplemented with the appropriate amino acid restored bacterial growth to wildtype levels (Fig. 3A). The apparent amino acid auxotrophy of these disruptants was associated with variable effects on *in planta* bacterial growth. When assessed by pressure infiltration of soil-grown plants, the *hisD* disruptant (*hisD::mTn5*) grew at least two logs less than wildtype, while the growth of *serB::mTn5* was reduced by 1.6 logs and *gltB::mTn5* showed no significant difference relative to wildtype (Fig. 2C). Spray inoculations revealed further growth impairments, including an approximately four-log reduction in the growth of the *serB* and *hisD* disruptants and 0.5–0.9 logs lower growth for *gltB::mTn5* (Fig. 2D). A different pattern was observed following the inoculation of seedlings grown in liquid media, where the *in planta* growth of *gltB::mTn5* and *hisD::mTn5* was reduced by approximately one log relative to wildtype *Pma* ES4326, while *serB::mTn5* grew 2.5 logs less (Fig. 3B). Bacterial growth in soil-grown plants could not be rescued by supplementing the inocula with millimolar concentrations of amino acids (data not shown), likely due to diffusion or metabolism of these compounds *in planta*. In the 96-well liquid assay, however, the growth deficit of all three disruptants could be ameliorated by the addition of amino acids (Fig. 3B).

Profiles of free amino acids in Arabidopsis leaf tissues have been determined in multiple studies, and it is interesting to note that glutamate is consistently found to be one of the most abundant amino acids, with serine present at intermediate concentrations, and histidine relatively scarce [28]–[31]. Similar relative abundances were observed in tomato apoplast fluids [32]. The collection of Arabidopsis apoplast extracts is not trivial, but some analyses have indicated that glutamate and serine are present at similar concentrations [28], [33]. At a general level, therefore, the extent to which the growth of amino acid auxotrophs is impaired can be linked to amino acid abundance *in planta*. If glutamate and serine are in fact equally abundant, the greater growth defect of the *serB* disruptant may result from the function of serine as a precursor for other amino acids such as glycine, cysteine, and tryptophan [34]. The scarcity of these three amino acids in apoplastic fluids [28], [32], [33] may render the available serine insufficient to satisfy all of the downstream metabolic requirements of *serB::mTn5* bacteria. The further reductions in bacterial growth observed for all three amino acid auxotrophs following spray inoculation suggest that free amino acids may be even more limited on leaf surfaces and/or that these amino acids are particularly important for overcoming pre-invasive immune responses such as stomatal closure.

The amino acid profile of Arabidopsis seedlings grown in liquid media is similar to that of adult plants [31], yet we found that the *serB* disruptant exhibited the most severe growth impairment in the liquid assay, while the disruption of *hisD* and *gltB* had less dramatic effects on growth. It is worth noting that the media used by Pratelli *et al*. [31] was supplemented with 1% (w/v) sucrose, and that sucrose supplementation can alter the amino acid profile of plant tissues [35]. It is also possible that the disruption of *serB* in *Pma* ES4326 has pleiotropic effects on metabolism and/or virulence that are exacerbated in the liquid assay. Nonetheless, the ability to rescue *in planta* bacterial growth in this assay by amino acid supplementation indicates that the capability to synthesize glutamate, histidine, and serine is critical for full virulence on Arabidopsis.

### Genes Associated with Other Metabolic Processes

**Figure 4:**
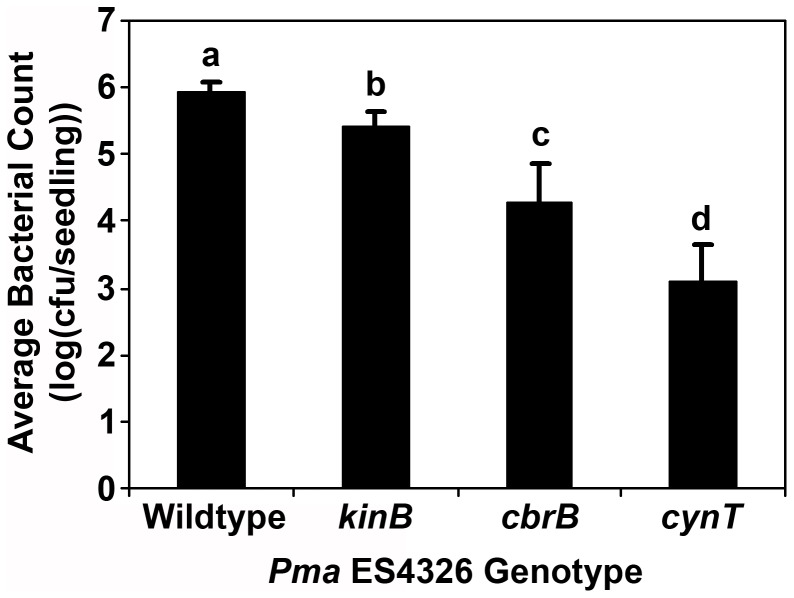
Growth in liquid-grown Arabidopsis seedlings of *Pma* ES4326 transposon disruptants whose growth in soil-grown plants is not impaired. Wells were inoculated with disruptants of the sensory box histidine kinase genes *cbrA* or *kinB*, or the carbonic anhydrase gene *cynT*. Bacterial populations were quantified at three days post-inoculation by dilution plating. Error bars reflect standard deviation of the mean of eight replicate samples. Letters above data points indicate statistical significance groups as determined by pairwise Student’s t-tests (α = 0.05). Three independent experiments were performed with similar results.

Three independent transposon insertions were identified in a homolog of *cynT*, which encodes a β-class carbonic anhydrase. This widely distributed enzyme catalyzes the interconversion of carbon dioxide (CO_2_) and bicarbonate ions (HCO_3_^-^), with proposed functions in the maintenance of intracellular pH and the supply of CO_2_/HCO_3_^-^ for enzymatic reactions [36]. Interestingly, the *in vitro* growth of *cynT::mTn5* in PMMF was similar to that of wildtype *Pma* ES4326, but was severely impaired when incubated in MSS media (Fig. 1C, D). Furthermore, bacterial growth in liquid-grown seedlings was dramatically reduced (Fig. 4). It was therefore surprising that this disruptant exhibited no such growth reduction in or on the leaves of soil-grown plants (Fig. 2E, F). Over the course of the *in vitro* growth assays in liquid media, the pH of both PMMF and MS media is consistently around 5.8, while MSS is generally pH 5.0–5.3 (T. Lo and D. Desveaux, unpublished data). This difference seems too subtle to have such dramatic effects on bacterial growth, especially since most apoplastic pH measurements fall between pH 5 and pH 6.5 [32], [37], [38]. Indeed, adjusting the pH of MSS to 5.8 did not mitigate the impaired growth of *cynT::mTn5* in this media (data not shown). It is therefore likely that the growth of *cynT::mTn5* is influenced by other factors such as the production of tissue- or developmental stage-specific molecules with which this disruptant is less able to contend.

Similar results were obtained with disruptants of two different sensory box histidine kinase genes (*cbrA*, *kinB*) that are members of two-component regulatory systems. In *P. aeruginosa*, CbrA and its cognate response regulator CbrB appear to monitor the intracellular carbon:nitrogen balance and adjust metabolism accordingly, such that *cbrAB* mutants cannot utilize a variety of organic compounds as a sole carbon source [39], [40]. More recently, Yeung *et al*. [41] noted that a *P. aeruginosa cbrA* mutant exhibited reduced swarming motility, enhanced biofilm formation, and increased *in vitro* cytotoxicity to human bronchial epithelial cells. The loss of *cbrAB* function in *P. fluorescens* caused pleiotropic defects in nutrient utilization and mRNA polyadenylation, and the mutation of CbrAB-regulated genes such as *pcnB* significantly reduced bacterial colonization of sugar beet seedlings [42], [43]. KinB was previously characterized as a negative regulator of alginate production [44] whose disruption compromises the virulence of *P. aeruginosa* in zebrafish embryos [45]. The disruption of *cbrA* in *Pma* ES4326 was associated with significantly reduced virulence in the liquid assay (∼1.5 logs less growth than wildtype), while the reductions observed for the *kinB* disruptant were more slight (∼0.5 logs) (Fig. 4). No such differences were observed in soil-grown plants for either disruptant (Fig. 2E, F) and *in vitro* growth in PMMF and MSS was similar to that of wildtype *Pma* ES4326 (Fig. 1G, H). Like the *cynT* disruptant, these results may be influenced by plant developmental stage and/or assay conditions.

### Conclusions

This screen was initiated as a high-throughput approach to the identification of virulence factors in the phytopathogen *Pma* ES4326. While many hits involved genes with previously characterized roles in Pseudomonad virulence, some novel components were also identified. We demonstrated that the ability of *Pma* ES4326 to synthesize specific amino acids strongly influences its proliferation in Arabidopsis leaf tissues, especially for amino acids like histidine that are present at very low concentrations in the host. Importantly, this result was confirmed by restoring wildtype levels of growth to these disruptants by supplementing the appropriate amino acid. Although we have described three amino acid auxotrophs here, the importance of other amino acids cannot be excluded, especially given the identification of additional auxotrophs in virulence screens using other *P. syringae* strains [2], [46], [47]. Approximately 12,600 *Pma* ES4326 disruptants were screened in this study, which for an organism with approximately 3,500 genes [8] would likely not be saturating. Furthermore, disruptants identified as hits from the primary screen were initially quantified in the media from the screening plates, and only those with near-wildtype populations were advanced for additional characterization. It is possible that some amino acid auxotrophs exhibited significant growth impairments and were excluded at this stage.

In addition to demonstrating the importance of amino acid biosynthesis for virulence, we also employed a number of different assays to reveal context-specific phenotypes for other disruptants of interest. In particular, disruption of the carbonic anhydrase gene *cynT* and the sensory box histidine kinase genes *cbrA* and *kinB* did not affect *Pma* ES4326 growth in soil-grown plants, but did significantly reduce bacterial virulence in liquid-grown seedlings. It is evident that the contribution of these specific genes to *Pma* ES4326 virulence is influenced by the developmental stage of the host plant and/or the use of a liquid media-based assay. Seedling infections can significantly affect later plant performance with regards to growth, yield, and even survival [48], [49], and therefore any insight into the genes required for such infections would be beneficial. The biological relevance of these genes for virulence on radish, the original host of isolation for *Pma* ES4326, is also worth further exploration. Future studies will also examine the chemical composition of media from liquid-grown seedlings in an effort to explain the compromised growth of *cynT::mTn5* in this media. Overall, we demonstrate the value of moving beyond traditional virulence assays for the identification and functional characterization of genes required for pathogen virulence.

## Supporting Information

Figure S1 **Expression of genes encoding flagellar regulatory proteins in wildtype** ***Pma*** **ES4326 and in a** ***fleN*** **disruptant (** ***fleN::mTn5*** **).** While *fleN* is a predicted negative regulator of flagellar biosynthesis, *fleQ* and *fliA* are thought to have positive regulatory functions. Expression values were normalized with the housekeeping gene *gyrB* as described in Methods S1 and reflect three technical replicates. Error bars represent standard deviation.
(TIF)

Figure S2 **Sample motility phenotypes of** ***Pma*** **ES4326 flagellar gene disruptants.** For each disruptant, two microlitres of a bacterial suspension (OD_600_ ≈ 0.1) was pipetted onto King’s B media containing 0.3% agar and motility assessed after two days of growth at 28°C. Visual (A) and quantitative (B) data are presented for a subset of the screening hits involving flagellar biosynthetic genes. Error bars reflect standard deviation of the mean of nine replicate samples. Letters above data points indicate statistical significance groups as determined by pairwise Student’s t-tests (α = 0.05). Two independent experiments were performed with similar results.
(TIF)

Figure S3 **Macroscopic symptoms following high-dose inoculation of Arabidopsis ecotype Eilenburg-0 with** ***Pma*** **ES4326.** Half of each leaf was inoculated with 5×10^7^ cfu/mL wild-type *Pma* ES4326, a type III secretion-deficient disruptant *(hrcN::mTn5*), or with disruptants of periplasmic glucan biosynthesis genes (*galU*, *mdoG*, *mdoH*). Images were captured at 20 hours post-inoculation. Asterisks denote leaves undergoing a programmed cell death response in the inoculated (upper) half of the leaf. Scale bar indicates 1 cm. Three independent experiments were performed with similar results.
(TIF)

Table S1 Primers used for gene expression analysis in Pma ES4326.
(DOC)

Methods S1 (DOC)
