# Supplementary figures and images for: A High-Throughput Forward Genetic Screen Identifies Genes Required for Virulence of Pseudomonas syringae pv. maculicola ES4326 on Arabidopsis
Source: PLoS One. 2012 Aug 1;7(8):e41461. doi: 10.1371/journal.pone.0041461 (PMC3409859; doi:10.1371/journal.pone.0041461)

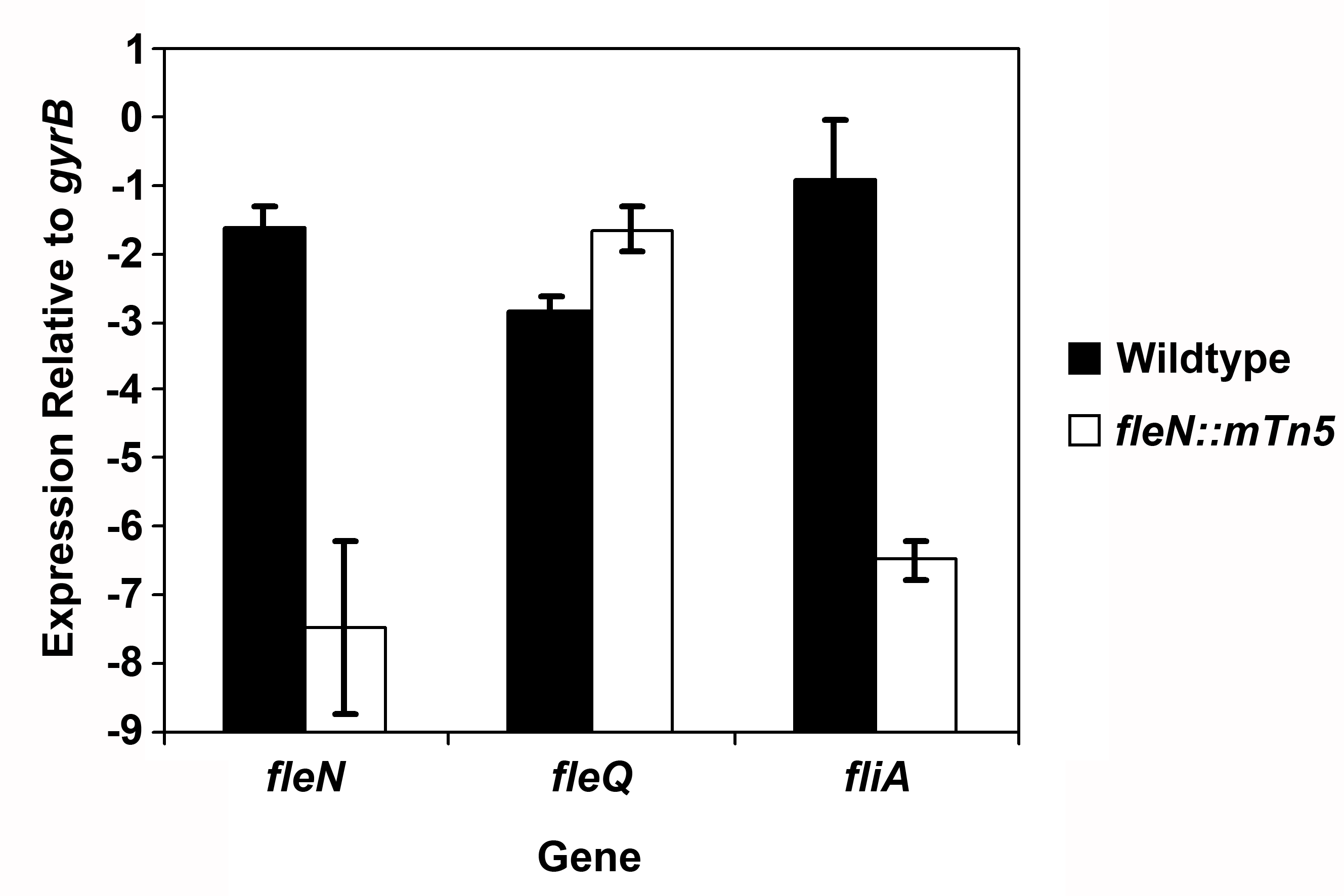

Supplement: Figure S1 — Expression of genes encoding flagellar regulatory proteins in wildtype Pma ES4326 and in a fleN disruptant ( fleN::mTn5 ). While fleN is a predicted negative regulator of flagellar biosynthesis, fleQ and fliA are thought to have positive regulatory functions. Expression values were normalized with the housekeeping gene gyrB as described in Methods S1 and reflect three technical replicates. Error bars represent standard deviation. (TIF) [file pone.0041461.s001.tif]

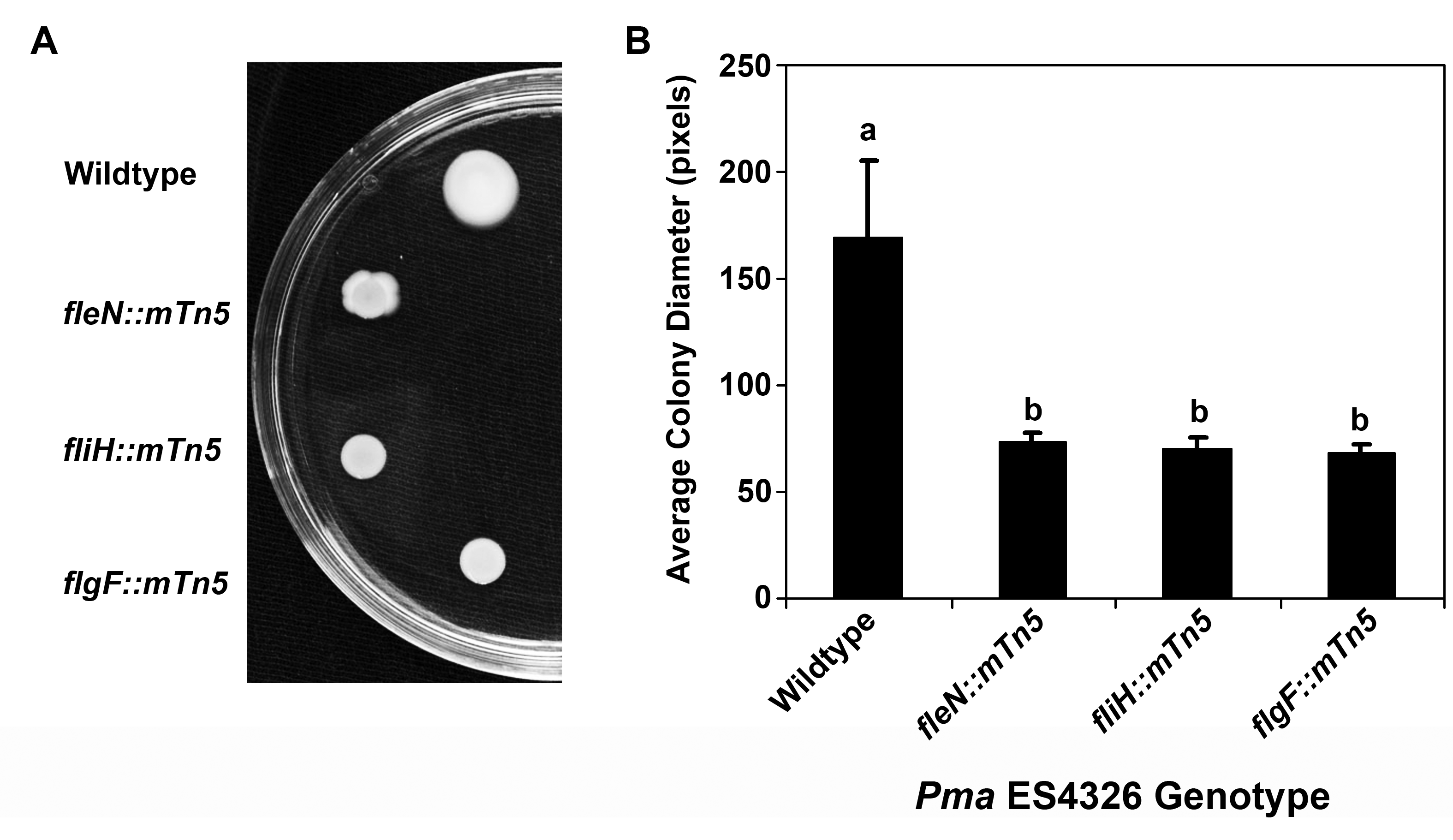

Supplement: Figure S2 — Sample motility phenotypes of Pma ES4326 flagellar gene disruptants. For each disruptant, two microlitres of a bacterial suspension (OD600 ≈ 0.1) was pipetted onto King’s B media containing 0.3% agar and motility assessed after two days of growth at 28°C. Visual (A) and quantitative (B) data are presented for a subset of the screening hits involving flagellar biosynthetic genes. Error bars reflect standard deviation of the mean of nine replicate samples. Letters above data points indicate statistical significance groups as determined by pairwise Student’s t-tests (α = 0.05). Two independent experiments were performed with similar results. (TIF) [file pone.0041461.s002.tif]

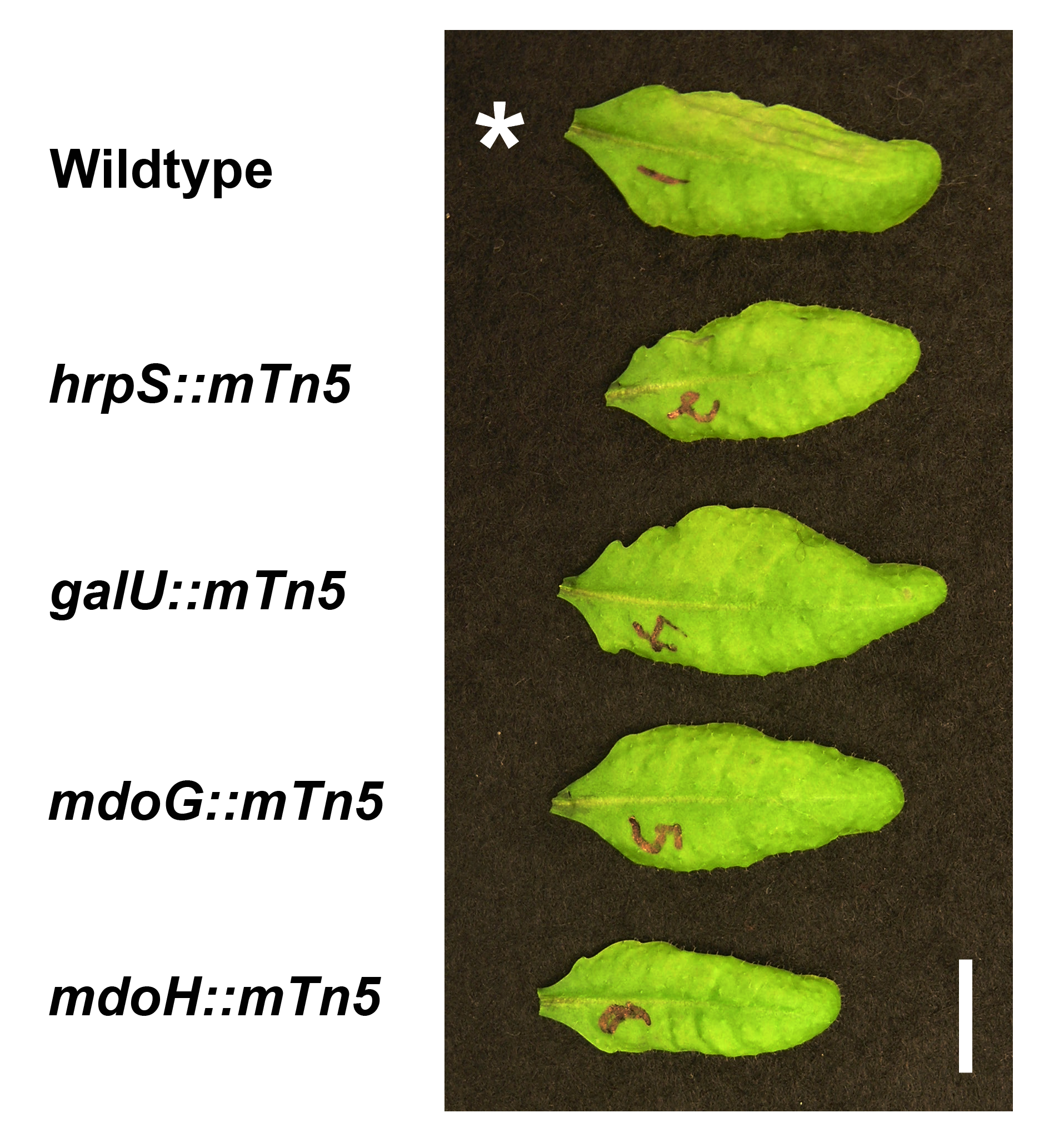

Supplement: Figure S3 — Macroscopic symptoms following high-dose inoculation of Arabidopsis ecotype Eilenburg-0 with Pma ES4326. Half of each leaf was inoculated with 5×10^7^ cfu/mL wild-type Pma ES4326, a type III secretion-deficient disruptant (hrcN::mTn5), or with disruptants of periplasmic glucan biosynthesis genes (galU, mdoG, mdoH). Images were captured at 20 hours post-inoculation. Asterisks denote leaves undergoing a programmed cell death response in the inoculated (upper) half of the leaf. Scale bar indicates 1 cm. Three independent experiments were performed with similar results. (TIF) [file pone.0041461.s003.tif]
