# Supplementary material for: A High-Throughput Forward Genetic Screen Identifies Genes Required for Virulence of Pseudomonas syringae pv. maculicola ES4326 on Arabidopsis
Source: PLoS One. 2012 Aug 1;7(8):e41461. doi: 10.1371/journal.pone.0041461 (PMC3409859; doi:10.1371/journal.pone.0041461)
Supplement: Table S1 — Primers used for gene expression analysis in Pma ES4326. (DOC) [file pone.0041461.s004.doc]

Table S1. Primers used for gene expression analysis in *Pma* ES4326

| **Primer Name**^a^ | **Sequence (5' to 3')** |
| --- | --- |
| gyrB_F(894) | GACGCGTAACCTGAACAACTAC |
| gyrB_R(1022) | TTGTCCTTGGTCTGCGAACTGAA |
| fleN_F(1) | ATGCATCCCGTACAGGTGATC |
| fleN_R(199) | GACCCTCGATCACATCGGCAAG |
| fleQ_F(752) | GTTGCCGATGCAGGTCAAACTGTTG |
| fleQ_R(899) | CGAAAGCTGCCGATCTCGATCATG |
| fliA_F(157) | TGATCGGTCTGCTTGAGGTCTCCAC |
| fliA_R(326) | CGAATTGCGTCACTGACCATGCGTG |

^a^Numbers in parentheses indicate the nucleotide positions at which primers anneal within the open reading frame of a given gene.
