# Supplementary material for: A High-Throughput Forward Genetic Screen Identifies Genes Required for Virulence of Pseudomonas syringae pv. maculicola ES4326 on Arabidopsis
Source: PLoS One. 2012 Aug 1;7(8):e41461. doi: 10.1371/journal.pone.0041461 (PMC3409859; doi:10.1371/journal.pone.0041461)
Supplement: Methods S1 — (DOC) [file pone.0041461.s005.doc]

**Methods S1**

For analyses of gene expression in *P. syringae*, overnight cultures were subcultured in King’s B media and incubated at 28ºC with shaking. When the cultures reached OD_600_ = 0.8, RNA extractions were performed using RiboPure-Bacteria reagents (Applied Biosystems, Streetsville, ON, Canada) and cDNA subsequently generated from 1.5 μg of DNaseI-treated total RNA using Superscript II reverse transcriptase (Invitrogen Canada Inc., Burlington, ON, Canada) and the primers described in Table S1. Real-time quantitative PCR was then performed with iQ SYBR Green Supermix (Bio-Rad Laboratories Ltd) in conjunction with the iCycler iQ real-time PCR detection system (Bio-Rad Laboratories Ltd). Following PCR, melt-curve analysis was performed to confirm the specificity of the amplification reaction. Relative transcript abundance was calculated using the ΔΔCt method [1], having verified similar amplification efficiencies for all primer sets. *P. syringae* transcriptional data were normalized to the housekeeping gene *gyrB*. The expression of a given gene relative to *gyrB* is calculated as the difference in qPCR threshold cycles (ΔCt = Ct_gene of interest_ - Ct_gyrB_). Comparisons between genotypes are calculated as the difference between ΔCt values (ΔΔCt). Since one PCR cycle represents a two-fold difference in template abundance, fold-change values are calculated as: 2^-ΔΔCt^.

**Reference**

1. Livak KJ, Schmittgen TD (2001) Analysis of relative gene expression data using real-time quantitative PCR and the 2^-ΔΔCT^ method. Methods 25: 402-408.
